# Tailoring Mesoporous Titania Features by Ultrasound-Assisted Sol-Gel Technique: Effect of Surfactant/Titania Precursor Weight Ratio

**DOI:** 10.3390/nano11051263

**Published:** 2021-05-11

**Authors:** Elvira Mahu, Cristina Giorgiana Coromelci, Doina Lutic, Iuliean Vasile Asaftei, Liviu Sacarescu, Valeria Harabagiu, Maria Ignat

**Affiliations:** 1 Laboratory of Materials Chemistry, Faculty of Chemistry, “Alexandru Ioan Cuza” University of Iasi, 11 Carol I Boulevard, 700506 Iasi, Romania; mahu.elvira@icmpp.ro (E.M.); doilub@uaic.ro (D.L.); ivasaftei@yahoo.com (I.V.A.); 2 Laboratory of Inorganic Polymers, “Petru Poni” Institute of Macromolecular Chemistry, 41 A, Grigore Ghica Vodă Aley, 700487 Iasi, Romania; livius@icmpp.ro (L.S.); hvaleria@icmpp.ro (V.H.); 3 Institute for Interdisciplinary Research—Science Research Department, “Alexandru Ioan Cuza” University of Iasi, Lascar Catargi Str. 54, 700107 Iasi, Romania; cristina.pastravanu.coromelci@gmail.com

**Keywords:** sol-gel technique, ultrasonation, titania, surfactant weight ratio, photocatalysis

## Abstract

A mesoporous titania structure has been prepared using the ultrasound-assisted sol-gel technique in order to find out a way to tailor its structure. The TiO_2_ obtained was compared to the same version of titania but synthesized by a conventional sol-gel method with the objective of understanding the effect of ultrasound in the synthesis process. All synthesis experiments were focused on the preparation of a titania photocatalyst. Thus, the anatase photocatalytic active phase of titania was proven by X-ray diffraction. Additionally, the ultrasonation treatment proved to increase the crystallinity of titania samples, being one of the requirements to having good photocatalytic activity for titania. The influence of surfactant/titania precursor weight ratio on the structural (XRD), textural (N_2_-sorption measurements), morphological (TEM), surface chemistry (FTIR) and optical properties (UVDR) was investigated. It was observed that the crystallite size, specific surface area, band gap energy and even photocatalytic activity was affected by the synergism occurring between cavitation effect and the surfactant/titania precursor weight ratio. The study yielded interesting great results that could be considered for further application of ultrasound to tailor mesoporous titania features via sol-gel soft template synthesis, against conventional sol-gel process.

## 1. Introduction

In the past decade, an important semiconductor with huge potential for applications in various areas of nanotechnology has caught researcher’s attention. Thus, titanium oxide proved to be attractive for important technological reasons (energy, environment, built environment, biomedicine) [1,2,3,4]. Because of its great photocatalytic properties, increasing attention has recently been focused on the simultaneous achievement of high specific surface area and the formation of crystalline mesoporous TiO_2_ frameworks. Nowadays, many researchers are still focused on mesoporous TiO_2_ photocatalyst because its textural properties promote the diffusion of reactants to the reactive sites, enhancing the photocatalytic activity [5,6,7]. This evolution has demonstrated that mesoporosity of TiO_2_ nanoparticles plays an important role in environmental protection.

Up to now, many physical methods have been used in the synthesis of titania nanoparticles [8]. Among them, the most common applied process is a solution-based one that is used for making inorganic networks of various nanomaterials through sol-gelation [9]. The sol-gel route is very attractive due to the ease of experimental setup to adjust the particles’ morphology by controlling the hydrolysis rate of titania alkoxides and condensation reactions of alcohol and water molecules [8].

In the last decade, ultrasounds have been applied in many synthesis procedures to develop various nanomaterials. Thus, ultrasonic-assisted synthesis proved to be an effective technique for generating nanoparticulate materials exhibiting attractive properties in a shorter time [10,11]. It is well-known that ultrasound has an enhanced chemical effect which is due to acoustic cavitation phenomena, leading to the rapid formation, growth and collapse of bubbles in liquid [12].

Recently, we applied ultrasonation to the sol-gel synthesis method of mesoporous titania nanoparticles. The synthesis process was empirically modeled and optimized using the response surface methodology (RSM), leading to a successful mesoporous titania structure [13]. The later experiments, conducted to new findings in this field of research, and further, the obtained results, will be presented.

In fact, this work describes a comparative study regarding sol-gel synthesis of a mesoporous titania structure and how ultrasonation-assisted synthesis affects the structure of mesoporous TiO_2_ nanoparticles. In the present report, the effect of the surfactant/titania source weight ratio is studied, and a comparison with non-ultrasonation process is given. To understand the exact function of structure-directing agents under ultrasound conditions in the proposed synthesis method, surfactant F127 of different weights was used. Thus, titania oxide materials with different characteristics have been obtained and characterized.

Because one of the current environmental issues is water pollution, and approximately 80% of the world’s population faces a high risk of contaminated water, titania materials are intensively studied as photocatalytic materials. Huge development of industry has led to an increase in the number of different dyes, pesticides, herbicides and fertilizers in groundwater and surface waters [14]. They have negative effects on human health, thus, millions of people die every year from diseases transmitted through unsafe waters [15]. The dyes are a considerable health preoccupation, because most of these compounds are potential or known human cancerogenics, even at low concentrations [15,16]. Among all developed photocatalysts, TiO_2_ is recognized as a top photocatalyst for the degradation of these harmful compounds [17]. Thus, the photocatalytic test results upon UV light will be shown comparatively and discussed, concerning the influence of the synthesis method and used surfactant/titania source weight ratios.

## 2. Materials and Methods

### 2.1. Materials

Titanium (IV) isopropoxide (C_12_H_28_O_4_Ti, purity ≈ 97%), Pluronic^®^ F-127 (tri-block copolymer of poly(ethylene oxide)-poly(propylene oxide)-poly(ethylene oxide)) (PEO_101_-PPO_56_-PEO_101_) (molecular weight 12,600 g/mol), and methylene blue (C_16_H_18_ClN_3_S · 3H_2_O) were purchased from Sigma Aldrich (Taufkirchen, Germany). Isopropyl alcohol (Iso-C_3_H_7_OH, ≥99.7%) and ethanol (C_2_H_5_OH, 99.3%) were purchased from S.C. Chemical Company S.A. (Iași, Romania). All products were analytical grade and used as received. In all experiments, ultrapure water produced with a PURELAB Ultra water purification system was used.

### 2.2. Preparation of Mesoporous Titania by Conventional Sol-Gel Method—A-Series

In a typical sol-gel synthesis procedure, titanium (IV) isopropoxide (TTIP) was used as titania precursor, and a non-ionic surfactant, Pluronic^®^ F127, as structure-directing agent (soft template). First, a clear solution containing the surfactant in a 50/50 (wt/wt. %) isopropyl alcohol–water mixture was obtained on a magnetic stir plate (A). Further, a well-known volume of TTIP was added dropwise through a syringe 21G needle, with a slow dripping rate of 1.8 mL/min, to the surfactant solution under continuous stirring. When the total volume of TTIP was added, the temperature of the stirring plate was raised up to 60 °C and left overnight at this working temperature under continuous stirring, allowing gel maturation. Thus, TTIP molecules hydrolyze, forming titanium hydroxide that polycondensate in TiO_2_ sol particles, being considered the start of gel aging.

$$\mathrm{Ti}{\left({\mathrm{OC}}_{3}H_{7}\right)}_{4}\overset{\begin{array}{c} \mathrm{hydrolysis}\\ \mathrm{condensation} \end{array}}{\to }\;[\mathrm{Ti}{\left(\mathrm{OH}\right)}_{4}]\;\overset{\begin{array}{c} \mathrm{drying}\\ \mathrm{calcination} \end{array}}{\to }{\mathrm{TiO}}_{2}$$

After 24 h of ageing, the resulted gel was centrifuged. The obtained white solid was washed thrice with ultrapure water and an additional time with ethanol, and left overnight in a dry oven at 50 °C. Then, the dried solid was calcined in a muffle furnace at 450 °C for 4 h, with a heating rate of 1 °C/min, to remove the surfactant molecules, leaving behind pores. The resulted titania samples prepared by the conventional stirring method were labeled as 5_A, 10_A, and 15_A, according to Table 1.

### 2.3. Preparation of Mesoporous Titania by Ultrasound-Assisted Sol-Gel Method—US-Series

As already described for conventional sol-gel synthesis, the same reactants were used in the ultrasound-assisted sol-gel synthesis of titania samples. The ultrasound-assisted sol-gel synthesis procedure has been reported by the authors in their previous work [13]. Thus, when the clear solution of F127 surfactant in 50/50 (wt/wt. %) isopropyl alcohol–water mixture was obtained, a horn-probe sonic tip (Sonics VCX-750 VibraCell Ultra Sonic Processor device, 750 W) working at 25% of amplitude with a pulsed ON/OFF cycle of 3/1 s was immersed. The working time for the sonic tip was set up for 1 h to allow TTIP to hydrolyze and isopropyl alcohol and water molecules to condensate. The working temperature was raised up to 60 °C and kept at this value during the ultrasonic treatment.

During the first 10 min of ultrasonation (US), the well-known volumes of TTIP were added dropwise to the surfactant solution, afterward leaving the mixture under ultrasound action for the remaining 50 min. Afterwards, the resulted white solid was separated by centrifugation, washed three times with ultrapure water, and lastly washed with ethanol and left to dry overnight at 50 °C. Further, the obtained powders were calcined in a muffle furnace at 450 °C for 4 h, with a heating rate of 1 °C/min. The resulted titania samples were labeled as 5_US, 10_US, and 15_US, as listed in Table 1.

### 2.4. Characterization of Prepared Mesoporous Titania

The characteristics of the prepared titania samples were investigated by various techniques such as X-ray diffraction (XRD), nitrogen-sorption measurements, transmission electron microscopy (TEM), Fourier transform infrared (FT-IR), and UV-Vis diffuse reflectance (UV-DR) spectroscopy.

XRD patterns were registered on a Shimadzu LabX XRD-6000 device (Shimadzu Co., Kyoto, Japan) with a scanning rate of 0.02°/min over a range of 20–80° 2*θ*, using CuKα radiation (*λ* = 1.5406 Å). The unit cell parameters were calculated using equation characterizing tetragonal symmetry:

(1) $$\frac{1}{d_{hkl}^{2}}=\frac{h^{2}+k^{2}}{a^{2}}+\frac{l^{2}}{c^{2}},$$

where *h*, *k*, and *l* are Miller’s indices and *d_hkl_* is the interplanar distance, deduced from Bragg’s equation:

(2) $$d_{hkl}=\frac{\lambda }{2\sin\;\theta }.$$

The crystallite size was estimated with the Scherrer formula:

(3) $$D_{Scherrer}=\frac{0.94\lambda }{\beta \times \;\cos\;\theta_{hkl}},$$

where *β* is the full width at the half maximum (*FWHM*) corresponding to the [*hkl*] plane [18]. The dislocation density, using the formula:

(4) $$\delta =\frac{1}{D_{Scherrer}^{2}},$$

The strain and the crystallite size were estimated using the equation:

(5) $$\beta =\beta_{\mathrm{size}}+\beta_{\mathrm{strain}}=\frac{K\lambda }{D_{W\;-\;H}\cos\;\theta }+4\varepsilon \;\sin\;\theta ,$$

where *β* is the actual broadening (FWHM), *θ*—the diffraction angle, *K*—the Debye–Scherrer constant, *λ*—the X-ray wavelength, D_W−H_—the crystallite size calculated by Williamson–Hall theory, and *ε*—the micro-strain. The registered XRD patterns allowed to calculate the crystallinity of the synthesized titania samples, defined as the ratio of “crystallinity peak area” ($A_{crystalline}$) and “total of crystalline and amorphous peak area” ($A_{crystalline+amorphous}$), using the formula:

(6) $$Crystallinity,\;\%=\frac{A_{crystalline}}{A_{crystalline+amorphous}}\times 100,$$

The pure nitrogen adsorption measurements at −196 °C (in liquid nitrogen) were performed on a NOVA 2200 Quantachrome instrument (Quantachrome Corporation, Boynton Beach, FL, USA). Prior to registration physisorption measurements, all physically adsorbed species were removed from the adsorbent surface by outgassing the samples under high vacuum at room temperature (according to IUPAC) [19] and also to ISO 9277 [20]. The dedicated software of instrument was used for the data fitting within the usual adsorption models (Brunauer–Emmet–Teller (BET) model for specific area determination and Barret–Joyner–Halenda (BJH) for pore size distribution). The total pore volume was estimated directly from the nitrogen adsorption-desorption isotherm at relative pressure of *P/P*_0_ = 0.95 [21].

The morphology of the synthesized titania nanoparticles was investigated by TEM microscopy, registering micrographs on a Hitachi HT7700 microscope (Hitachi High-Technologies Corporation, Tokyo, Japan) equipped with a Bruker XFlash 6 EDS detector operated at 120 kV in high contrast mode. The samples were supported on copper grids by dropping the ethanol dispersion of powdered samples. Finally, before the measurements, the copper grids were kept in a dry oven at 50 °C overnight. The size distribution of titania nanoparticles measured on the resulted TEM micrographs were performed by using ImageJ software.

Fourier transform infrared (FTIR) spectroscopy was used to investigate the surface chemistry of the prepared titania samples. The spectra were recorded on a Bruker Vertex 70v FTIR spectrometer (Bruker Optics, Ettlingen, Germany), resolution of 2 cm^−1^, in the range of 4000–400 cm^−1^ by KBr pellet technique.

The UV-Vis diffuse reflectance spectra (UV-Vis-DRS) were recorded on a Shimadzu 2450 device (Shimadzu Co., Kyoto, Japan) equipped with an integrating sphere reflectance accessory. The measurement was performed by placing the sample in front of the incident light window and concentrating the light reflected from the sample on the detector using a sphere with a barium sulfate-coated inside.

### 2.5. Photocatalytic Performance Evaluation

The dye photodegradation tests were performed in batch open pots containing aliquots of aqueous methylene blue (MB) solutions (50 mL), under magnetic stirring. The irradiation was made with UV lamps *(λ_max_* = 365 nm, 8 W) placed 15 cm above the pots (covered with black, opaque textile covers). A stock solution of dye was prepared for a set of experiments and kept in the dark. Before the reaction, the working solution of 50 ppm was prepared in a flask of 250 mL and mixed in the dark for 30 min to reach the adsorption equilibrium, and then the UV lamp was switched on. Time zero on the dye degradation graphs was considered the moment of the lamp start; thus, the adsorption process is represented in the −30 and 0 min interval. Samples of about 3 mL were taken with a syringe at 0, 5, 10, 15, 20, 30, 45, and 60 min, filtered through membrane filters with pores of 0.45 microns to separate the photocatalyst powder, and the concentration of the clear dye solution was determined by UV-Vis spectrometry (on a Shimadzu 2450 spectrophotometer). MB has a strong main peak in the visible region of the UV-Vis spectrum at 668 nm, and its concentration can be measured on this basis. The dye molecule decomposition yield was measured as the decolorization extent. The percentage of dye removal was calculated with the equation:

(7) $$Dye_{removal}\left(\%\right)=\frac{c_{0}-c_{t}}{c_{0}}\times 100,$$

where $C_{0}$ and $C_{t}$, respectively, are the values of MB concentrations initially and at time t.

Photonic efficiency was determined using the following equation [22]:

(8) $$\mathrm{Photonic}\;\mathrm{efficiency}\left(\%\right)=\frac{\mathrm{reaction}\;\mathrm{rate}\;\left({\mathrm{mol}\;m}^{-2}{\;s}^{-1}\right)}{\mathrm{photon}\;\mathrm{rate}\;\left({\mathrm{Einstein}\;m}^{-2}\;s^{-1}\right)}\times 100,$$

The reaction rate calculations led to the best fit with the second-order reaction. The irradiance of the used UV lamp was measured with a HD2102.1 Photo-Radiometer.

## 3. Results and Discussion

### 3.1. XRD Measurements

**Figure 1:**
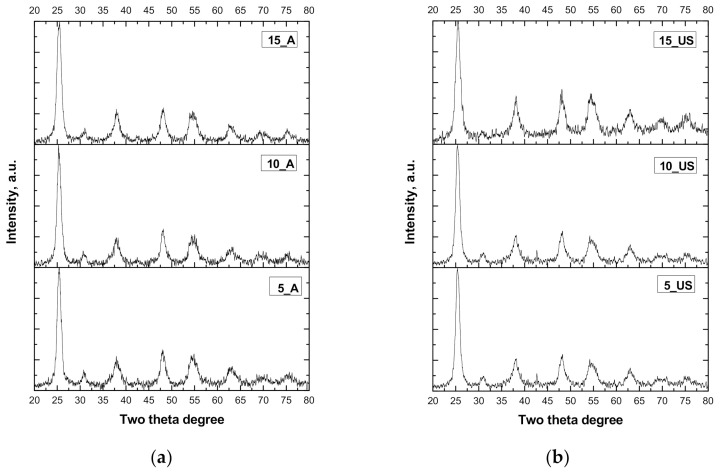
XRD patterns of the samples: (**a**) mesoporous TiO_2_ obtained by ultrasound-assisted synthesis; (**b**) mesoporous TiO_2_ obtained by stirring synthesis.

According to XRD patterns, all three samples exhibited a dominant anatase-phase of TiO_2_, proven by the diffraction peaks displayed in Figure 1, which are associated with the planes indexed as [101], [004], [200], [105], [211], [204], and [116], each corresponding to 2θ = 25.4, 38, 48, 54, 54.8, 62.5, and 68.8, respectively [23]. It is worth underlining that the structure type was not changed and the phase purity did not depend on the preparation procedure or on the different experimental ratios of used reactants. All synthetic experiments were conducted to the formation of anatase phase (according to JCPDS no. 01-089-4203), this being typical for preparing titania by hydrolysis-condensation procedure.

The registered XRD patterns indicate that the crystallization process strongly depended on the preparation procedure (see Table 2). Thus, the ultrasound treatment conducted to a more crystalline structure and well-defined particles compared to those obtained by a simple stirring method. This is indicated by the relatively smooth shape of the graphs of the US sample series (Figure S1). On the contrary, the shapes of the diffractograms were highly noisy in the case of A sample series. This conclusion is also supported by the calculated percentages of the crystallinity, which are given in Table 2. As crystallinity plays an important role in the photodegradation process of organics, the obtained results were analyzed from the photocatalytical point of view and compared US-series with A-series. As observed, the A-series of titania samples had crystallinity in the range of 82.5–83.9%, while the US-series exhibited crystallinity between 92.1 and 93.7%. This is a great finding, pointing out that the ultrasonation treatment led to more crystalline titania samples, enhancing it by about 9.8%. This fact is advantageous for the prevention of electron and hole recombination during the photocatalytic process, making the photocatalyst more efficient [24].

As the anatase has tetragonal symmetry [18], its unit cell parameters and interplanar distance (d_101_) were calculated and are summarized in Table S1 (Supplementary Materials). No trend was observed in the A-series of titania samples, whilst US-series exhibited a clear decrease of unit cell parameter (a_0_) as well as interplanar distance (d_101_) with increasing the surfactant/titania source weight ratio. Furthermore, crystallite size was estimated with the Scherrer formula, and the obtained values are presented in Table 2. As observed, different crystallite sizes in the range of 5–10 nm were obtained. Thus, the higher the surfactant concentration used, the smaller the formed crystallites. This result suggests that when surfactant concentration was higher, the template micelles competed to grab as much of the titanium precursor around them as possible. Furthermore, in order to describe the deficiency in the nanoparticles which measures the number of defects and vacancies in the crystal, considering the crystallite size $D_{Scherrer}$, the dislocation densities (δ) for the synthesized titania samples were calculated (Table 2). Looking at the obtained results, it was observed that with increasing surfactant/titania source weight ratio, the structural parameters followed a trend in both cases of A-series and US-series. Comparing the dislocation densities of both series, one can conclude that the US-series exhibited lower values than the A-series. This is an indication of increased plastic deformation in titania materials prepared using ultrasonation.

The Scherrer equation considers only the effect of crystallite size on the XRD peak broadening and does not tell anything about the intrinsic strain. Williamson–Hall (W-H) plot analysis was performed, allowing to determine the effect of the strain induced by XRD peak broadening [25]. Thus, by plotting β cos θ vs. 4 sin θ, the W–H crystallite size through the intercept and the strain by the slope have been determined, and the obtained values are presented in Table 2. The crystallite sizes calculated by Williamson–Hall analysis showed crystallite sizes about 2.1 nm smaller than those estimated with Scherrer equation. The variation of lattice strain with the surfactant/titania source weight ratios, and the influence of ultrasounds on lattice strain, were also observed (Table 2). As all prepared titania samples showed a negative value (*ε* < 0), meaning that the strain in this material was compressive, and the ultrasonation gave rise to more compressive strain. The negative strain suggests the reduction of defects on the titania surface, releasing the strain and relaxing the lattice [26].

### 3.2. Textural Characteristics Investigation

**Figure 2:**
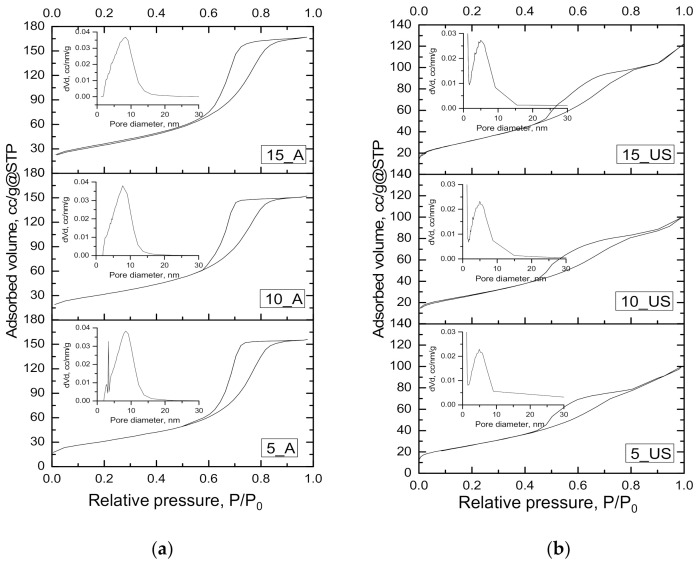
Nitrogen adsorption-desorption isotherms for titania samples: (**a**) A-series; (**b**) US-series, and the corresponding BJH pore size distributions calculated using adsorption branch (inset).

The textural features of the prepared titania samples were evaluated using the registered nitrogen adsorption-desorption isotherms at 77 K (Figure 2) and by the data processing using several adsorption models. The resulted isotherms for the A-series had mostly type IV isotherm shapes (Figure 2a) according to IUPAC classification [19,27]. Regardless of the used surfactant/titania precursor weight ratio, the isotherms of the A-series were similar to each other, being characteristic to mesoporous materials. The same was true for the US-series. An important feature of the isotherms is the final saturation plateau [19], which was characteristic only of the A-series of titania samples, indicating the fulfilling of mesopores, excluding the presence of macroporosity—the external porosity is very small [28]. The isotherms of US-series showed a rise up in adsorbed amount near saturation, being visible in the range of relative pressures of *P/P_0_* = 0.9–1, which is associated with condensation in interparticle voids [27].

For both investigated series of titania samples, appearance of a hysteresis loop was observed. According to IUPAC classification [19], the hysteresis loop is of H2 type, suggesting that titania materials have a complex, interconnected pore structure. Usually, such pore structures are characterized by a networking effect, characteristic of porous glass, exhibiting cavities connected by constrictions [29]. Thus, the formation of H2 hysteresis loop seems to be the signature of percolation in a narrow range of pore necks or to cavitation-induced evaporation. The A-series exhibited an H2 loop (Figure 2a) that was assigned to the a wide distribution pore bodies with a narrow neck size distribution (ink-bottle pores), while the US-series showed a H2 loop (Figure 2b) which was associated with a narrow distribution of pore bodies with a wide neck size distribution [19,21]. These assumptions are in line with the data issued from the BJH pore size distributions calculated using the data of adsorption branch of isotherm (Figure 2 (inset)) and of desorption branch (Figure S2—Supplementary Materials). These results show wide pore size distribution of A-series compared to that of US-series, while the mean pore diameter was larger for the A-series in comparison with that of the US-series. For the US-series, the presence of a dual pore system was highlighted by the BJH pore size distribution calculated using desorption branch of the isotherm (Figure S2—Supplementary Materials). Along with the hysteresis type, this is one more indication of a complex network of interconnected pores of ink-bottle shapes.

The BET equation [19] allowed to calculate the specific surface, while the total free pore volume was estimated from the adsorption branch of isotherm at relative pressure of *P/P_0_* = 0.95 (both are given in Table 3). Thus, it can be observed that the ultrasonation treatment led to a diminished specific surface area (by about 10%) and total pore volume (by about 35%). However, the textural parameters still had great values to be considered for adsorption-photocatalytic processes, allowing the surface to guest other species. Thus, the prepared titania samples exhibited specific surface areas above 100 m^2^ g^−1^ that increased with increasing surfactant/titania source weight ratios. The higher ratios of surfactant/titania source were favorable in preparing titania samples with higher free pore volumes. This was most probably due to the initial accommodation of a larger number of surfactant molecules auto-assembling in micelles, around which the rigid titania network is formed. It seems that an expansion of the titania structure occurred, which is supported as well by an increased specific surface area. On the contrary, Benkacem and Agoudjil investigated the effect of the cetyltrimethylammonium bromide/titanium alkoxide ratio on textural properties of titania, and they found that a ratio increase leads to diminished textural parameters [30]. It is essential to mention that during the conventional stirring (A-series), the mean pore diameter decreased with increasing surfactant/titania source weight ratios, while ultrasonation had an opposite trend, with a slight increase of the mean pore diameter as the surfactant/titania source weight ratio increased. As the desorption isotherms did not return same way as adsorption isotherm, occurring a delay in desorption of N_2_ from pores, desorption data were exploited, and the mesopore sizes were calculated by applying the BJH model. Thus, the corresponding pore size distributions, depicted in Figure S2, showed uniform distribution in the case of A-series, while a very interesting finding was the non-uniform pore size distribution of the US-series, indicating a bimodal porous nature of titania samples (*D_pore(BJH des)_*, Table 3).

### 3.3. Morphology Investigation by TEM

**Figure 3:**
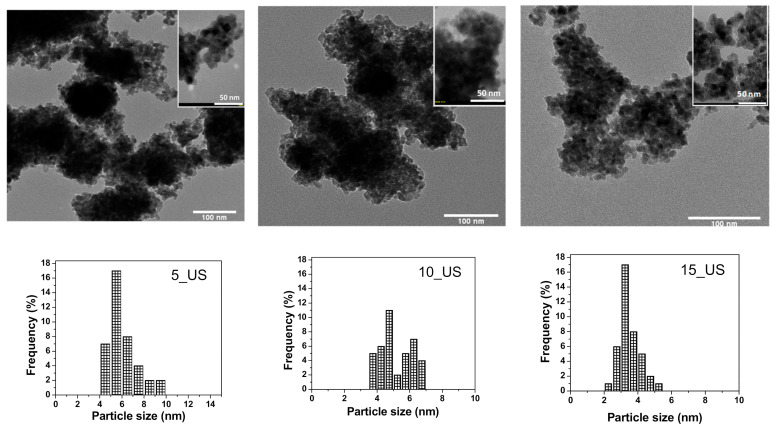
TEM images acquired on the US-series of titania samples, and corresponding particle size distribution.

**Figure 4:**
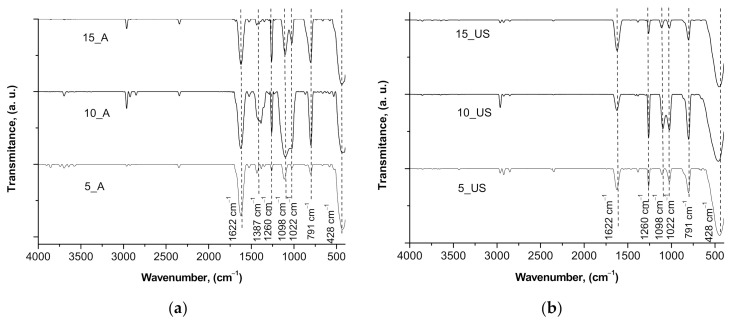
FTIR spectra registered for the (**a**) A-series and (**b**) US-series titania samples.

As the ultrasounds proved to lead to a titanium oxide nanostructure with structural and textural features comparable to those of their homologues synthesized by the conventional stirring method, TEM images were captured and are presented in Figure 3. All images show the particulate morphology for all titania samples of the US-series, looking very similar to each other. All three nanopowders were made up of pseudospherical particles, slightly associated in clusters. The TEM images were used to measure the sizes of the particles, with microscopic image processing software ImageJ, allowing to draw the particle size distributions (Figure 4, under each TEM image). As observed, the mean particle diameter determined using TEM images was closer to the crystallite size calculated using Williamson–Hall theory, being in good agreement with the XRD results.

### 3.4. Surface Chemistry and Optical Properties of the Synthesized Titania Samples

The surface chemistry of the synthesized mesoporous titania samples was investigated by FT-IR analysis (Figure 4). Significant hydroxyl groups on the surface of TiO_2_ nanoparticles could be observed. In addition, the crystallites size could result in the broadness of IR peaks. Thus, the peak at 1622 cm^−1^ observed in all spectra corresponded to hydroxyl groups anchored on the titania surface (bending modes of Ti–OH). Additionally, both titania samples series exhibited peaks at 1260 cm^−1^, 1098 cm^−1^, and 1022 cm^−1^, which could come from Ti–O–C bonds [31], suggesting the presence of C-species on titanium dioxide surface. The peaks appearing in the range 900–400 cm^−1^ were contributions from O–Ti–O bonding in anatase morphology [32,33], indicating the complete removal of all organics after annealing at 450 °C. However, only A-series showed the peak at 1387 cm^−1^ which is related to Ti–O modes [34]. Therefore, most probably, it was the reason for the higher dye adsorption for the A-series compared to that of the US-series determined further during photocatalytic experiments.

**Figure 5:**
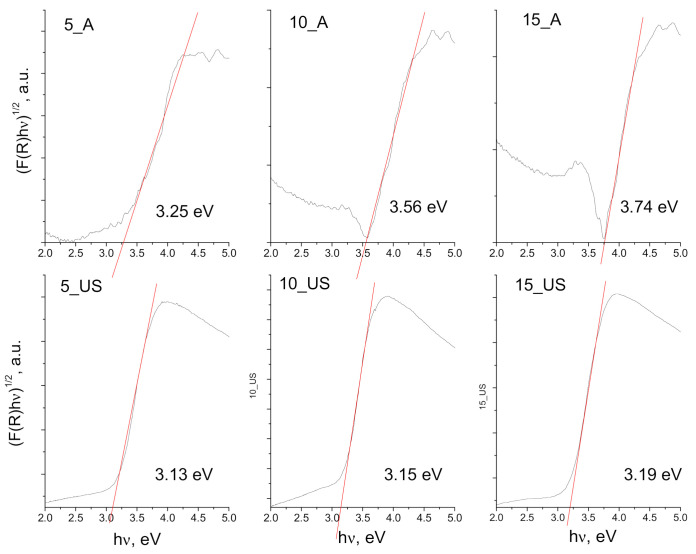
Tauc plots for synthesized titania samples (A-series and US-series) and corresponding band gap energies.

UV-Vis diffuse reflectance spectra (UV-Vis-DRS) for the prepared titania samples were registered, allowing to determine the band gap energy. According to the Tauc theory [35], band gap energies for each sample were estimated by extrapolating the linear part of Tauc plot, as shown in Figure 5, respectively, the dependence of the Tauc function ${\left(\alpha h\upsilon \right)}^{1/2}$ vs. hυ [36]. The band gap energies for A-series of titania samples were found to be 3.27, 3.61, and 3.76 eV, respectively, while for the US-series, the determined band gap energies were smaller, 3.13, 3.15, and 3.19 eV (the energy values being between anatase and rutile, but closer to *Eg* value of anatase, reflecting the fact that anatase polymorphs dominate over rutile phase) [37]. The A-series titania samples all had band gap energies that exceeded 3.2 eV, most probably due to the lattice deformation by an axial strain [38]. A clear trend was observed for the evolution of the band gap energy: an increase with the increase of surfactant/titania source weight ratios occurred in both cases, conventional aging and utrasound-assisted synthesis.

### 3.5. Photocatalytic Activity Investigation for Methylene Blue Degradation

**Figure 6:**
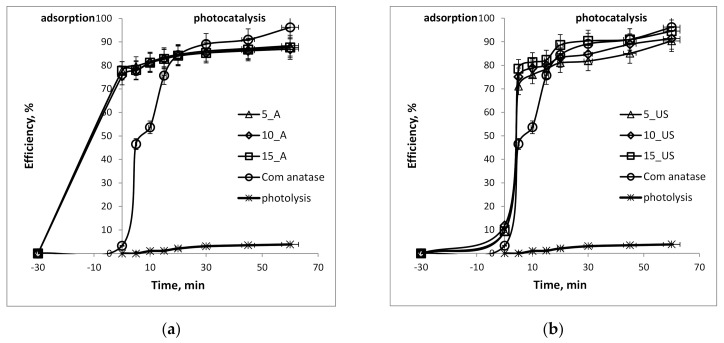
Photodegradation efficiency of the synthesized titania nanoparticles for degradation of MB under UV light: (**a**) TiO_2_ samples of A-series; (**b**) TiO_2_ samples of US-series.

As one of the key features of titanium oxide is its band gap, the potential of the prepared photocatalysts can be estimated. Thus, a model molecule was considered, and photocatalytic performance was determined. Before investigating the photocatalytic activity of the synthesized samples, the transformation of MB (20 mg L^−1^) at natural pH (5.5) and 20 °C under UV irradiation (365 nm) was carried out. It was observed experimentally that direct photolysis of substrates was very slow, showing no significant photodegradation of MB. The photocatalytic activity of titania samples of both series were compared, also with the performance of commercial TiO_2_ nanoparticles (21 nm, anatase phase from Sigma Aldrich, labeled in the present work as Com anatase). The MB removal efficiency is displayed in Figure 6.

The photocatalyst behavior in contact with MB solution was remarkable in terms of fast removal (in only 60 min) of the dye in both cases. However, a big difference between TiO_2_ A-series and TiO_2_ US-series could be observed. Thus, the removal rate over 77% in the case of A-series was due to the physical adsorption of MB on the TiO_2_ surface, while its photocatalytic degradation seemed to be of about 88%. The enhanced MB sorption on TiO_2_ photocatalyst synthesized in a conventional stirred sol-gel synthesis was most probably due to the higher specific surface area, larger pore diameter and pore volumes that are able to host organic molecules. In the case of the US-series, this ratio was completely different: the physisorption was about 10%, while the photocatalysis was over 90%. The last finding was most likely due to the photocatalytic activity of the commercially available anatase nanoparticles, which were tested as reference photocatalyst in the degradation process of MB dye. On the other hand, a great photocatalytic capacity of the US-series in the first 5 min of the photocatalytic experiment, its value far exceeding the photocatalytic capacity of the commercial anatase nanoparticles. It almost doubled in the first 5 min, but further achieved the same performance as commercial anatase nanoparticles. The calculated percentages for the adsorption and photocatalytic processes, as well as degradation capacities of the tested titania samples, are given in Table 4, comparatively.

**Figure 7:**
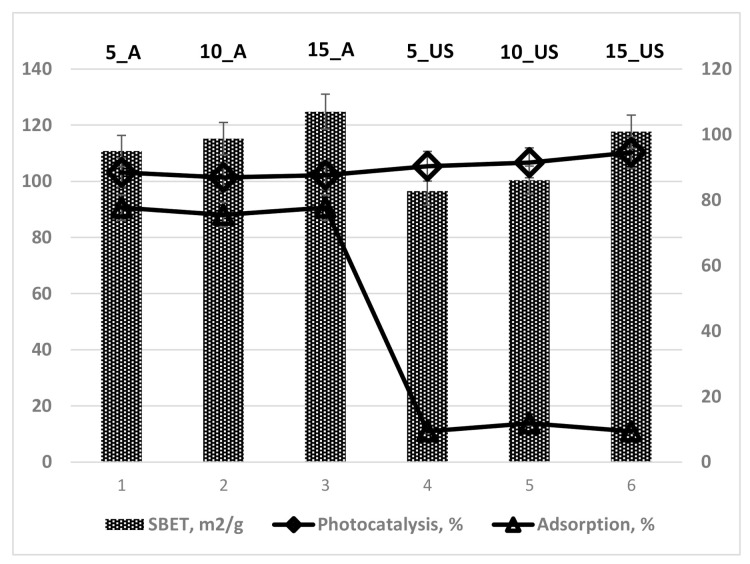
Correlation of photocatalytic activity and specific surface area for TiO_2_ samples prepared.

By correlating the photocatalytic activity of the synthesized titania samples with their specific surface areas, the plot in Figure 7 could be drawn. It can be observed that the US-series of titania samples exhibited specific surface area reduced by about 10% compared to that of the A-series. On the contrary, the adsorption did not follow the same trend as would be expected, the reduction being around 85% of the adsorption on the US-series compare to that of the A-series. Thus, the higher surface areas can be associated with higher adsorption of MB from solution for the A-series, while the US-series of titania samples showed very low adsorption. In contrast, the overall photocatalytic activity was higher for the US-series, even they exhibited lower specific surface areas and adsorption capacity, as well as photonic efficiency (Table 4).

## 4. Conclusions

Mesoporous titania was prepared by hydrolysis-condensation of an organic titanium salt, TTIP, by simple conventional stirring. By applying an ultrasonation treatment to the same reaction mixture, mesoporous titania nanoparticles could be obtained in a shorter time. The resulted powdered samples consisted of highly porous anatase phase, where the crystallite size calculated from XRD was the same as the nanoparticle size estimated from TEM. With an increase of surfactant concentration, a slight increase of crystallinity was observed. The same trend was also observed for specific surface area, band-gap energy, and even for the photocatalytic activity of the US-series of titania samples. The mean diameter of US-generated crystallites of TiO_2_ deduced from XRD analysis was found to be in the range of 5.1–7.2 nm, the size being achieved by decreasing the surfactant/titania source weight ratio. This result is in good agreement with TEM and UVDR analysis, resulting in enhanced photocatalytic activity over that of TiO_2_ A-series, and being comparable with the photocatalytic activity of commercially available anatase nanoparticles (Sigma Aldrich, 21 nm, 41.6 m^2^/g). Their textural features were greater than those of commercial anatase nanoparticles, making it advantageous for concentrating dye molecules around photocatalytic nanoparticles. Thus, by introducing ultrasonation, a lesser degree of agglomeration could be obtained, and the characteristics of mesoporous titania could be successfully tailored by varying the surfactant/titania precursor weight ratio in an ultrasound-assisted sol-gel synthesis procedure.

## Figures and Tables

**Table 1:** Molar ratios of the reactants used.

Sample Label		Used Molar Ratio of Reactants			
Conventional Stirring	Ultrasound- Assisted	TTIP, ×10^−2^	F-127, ×10^−4^	H_2_O	Iso-C_3_H_7_OH
5_A	5_US	4.9	3.3	2.3	1
10_A	10_US	4.9	6.6	2.3	1
15_A	15_US	4.9	9.9	2.3	1

**Table 2:** Structural features of the synthesized porous titania derived from XRD data.

Sample	*D_Scherrer_* (nm)	*D_W–H_* (nm)	*δ,* ×10^−2^ (nm^−2^)	*ε* (×10^−3^)	*Crystallinity, %*
5_A	9.6 ± 0.5	7.8 ± 0.8	1.077	−0.9	82.5
10_A	9.4 ± 0.5	8.0 ± 0.8	1.138	−0.8	83.9
15_A	8.6 ± 0.5	6.5 ± 0.8	1.349	−2.3	83.0
5_US	9.5 ± 0.5	7.2 ± 1.1	1.100	−1.6	92.1
10_US	8.8 ± 0.5	7.0 ± 1.1	1.306	−1.4	92.4
15_US	8.5 ± 0.5	5.1 ± 1.1	1.400	−3	93.7

*D_Scherrer_*—calculated crystallite size using Sherrer formula; *D_W–H_*—calculated crystallite size using Williamson–Hall theory; *δ*—dislocation density; *ε*—lattice strain.

**Table 3:** Textural features derived from N_2_-sorption isotherms of the synthesized porous titania.

Sample	*S_BET_,* m^2^/g	*D_pore(BJH ads)_*, nm	*D_pore(BJH des)_*, nm	*V_t_,* cm^3^/g
5_A	110.8	6.82	6.90	0.241
10_A	115.2	6.47	6.42	0.235
15_A	124.8	6.55	6.45	0.259
5_US	96.5	4.95	3.90; 4.43	0.151
10_US	100.4	5.01	3.90; 4.45	0.141
15_US	117.7	5.14	3.89; 4.72	0.190

*S_BET_*—specific surface area calculated using Brunauer–Emmet–Teller theory; D*_pore(BJH ads)_*—the mean pore diameter estimated from BJH-pore size distribution calculated using adsorption branch of isotherm; D*_pore(BJH des)_*—the mean pore diameter estimated from BJH-pore size distribution calculated using desorption branch of isotherm; *V_t_*—the total pore volume estimated from the adsorbtion branch at relative pressure of *P*/*P*_0_ = 0.95.

**Table 4:** Photocatalytic performance of the synthesized porous titania samples for MB degradation under UV-light.

Sample	Adsorption, %	Photocatalysis, %	*q_e_*, mg/g	Apparent Constant Rate, 10^−4^ s^−1^	Photonic Efficiency, %
5_A	77.7	88.4	55.3	4	11.9
10_A	75.5	86.9	54.4	4	15.1
15_A	77.7	87.6	54.8	4	13.5
Com anatase	3.3	96.2	60.1	12	19.7
5_US	9.4	90.3	56.5	10	20.3
10_US	11.8	91.4	57.1	12	19.7
15_US	9.4	94.5	59.1	19	12.9

## Supplementary Materials

The following are available online at https://www.mdpi.com/article/10.3390/nano11051263/s1. Figure S1: XRD patterns of titania samples prepared using the molar ratio of reactants TTIP:F-127:H_2_O:Iso-C_3_H_7_OH = 4.9 × 10^−2^:3.3 × 10^−4^:2.3:1 by stirring procedure (A) and ultrasound-assisted synthesis (US), Figure S2: Corresponding BJH pore size distributions isotherms for titania samples: (a) A-series; (b) US-series, calculated using desorption branch of isotherm, Table S1: Structural features of the synthesized porous titania samples derived from XRD patterns.
