# Supplementary material for: Tailoring Mesoporous Titania Features by Ultrasound-Assisted Sol-Gel Technique: Effect of Surfactant/Titania Precursor Weight Ratio
Source: Nanomaterials (Basel). 2021 May 11;11(5):1263. doi: 10.3390/nano11051263 (PMC8150942; doi:10.3390/nano11051263)
Supplement: Supplementary file 1 [file nanomaterials-11-01263-s001.zip › nanomaterials-1181966 SI - final.pdf]

Supplementary Materials

Tailoring Mesoporous Titania Features by Ultrasound-Assisted Sol-Gel Technique: Effect of Surfactant/Titania Precursor Weight Ratio

Elvira Mahu, Cristina Giorgiana Coromelci, Doina Lutic, Iuliean Vasile Asaftei, Liviu Sacarescu, Valeria Harabagiu and Maria Ignat

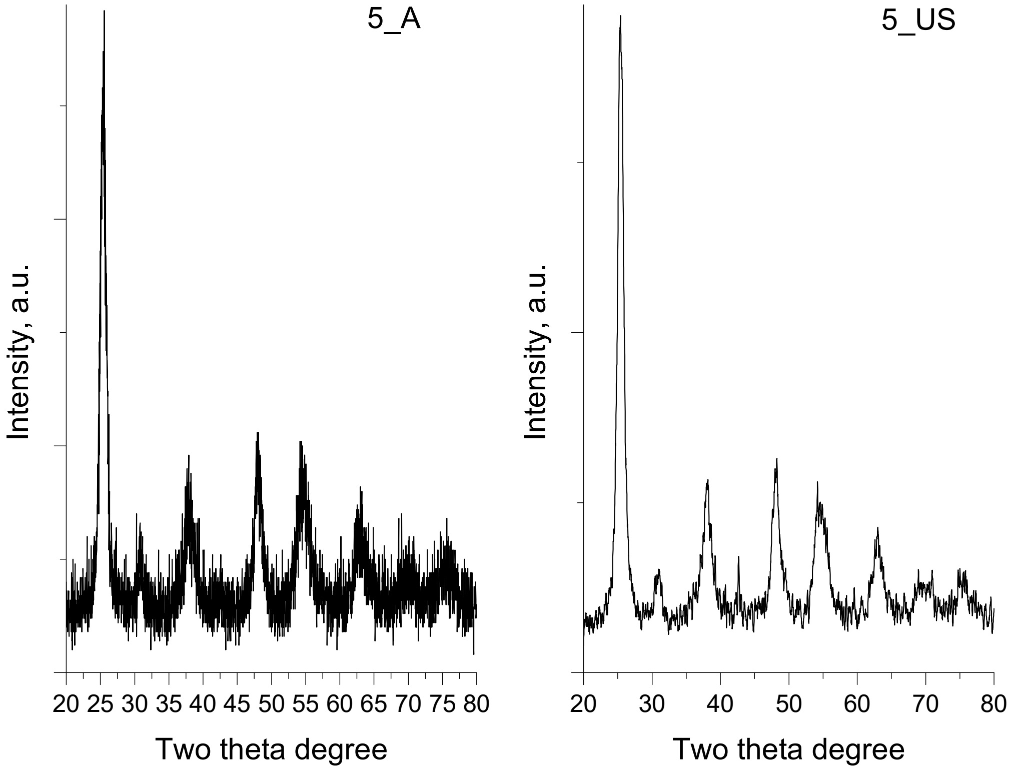

Figure S1. XRD patterns of titania samples prepared using the molar ratio of reactants TTIP:F-127:H_2O:Iso-C_3H_7OH = 4.9 × 10^{-2}:3.3 × 10^{-4}:2.3:1 (5) by stirring procedure (A) and ultrasound-assisted synthesis (US).

Table S1. Structural features of the synthesized porous titania samples derived from XRD patterns.

Sample	2θ[101], °	d[101], nm	*a₀*, nm
5_A	25.48	5.589	6.454
10_A	25.26	5.863	6.770
15_A	25.48	5.499	6.350
5_US	25.38	5.376	6.208
10_US	25.52	5.135	5.929
15_US	25.46	4.252	4.910

$2\theta_{[101]}$—diffraction angle of [101] crystallographic plane; $2\theta_{[004]}$—diffraction angle of [004] crystallographic plane; $d_{[101]}$—interplanar distance calculated from [101] peak; a_0—unit cell parameter; $D_{pore(BJH\ des)}$—pore diameter estimated from desorption branch of isotherm.

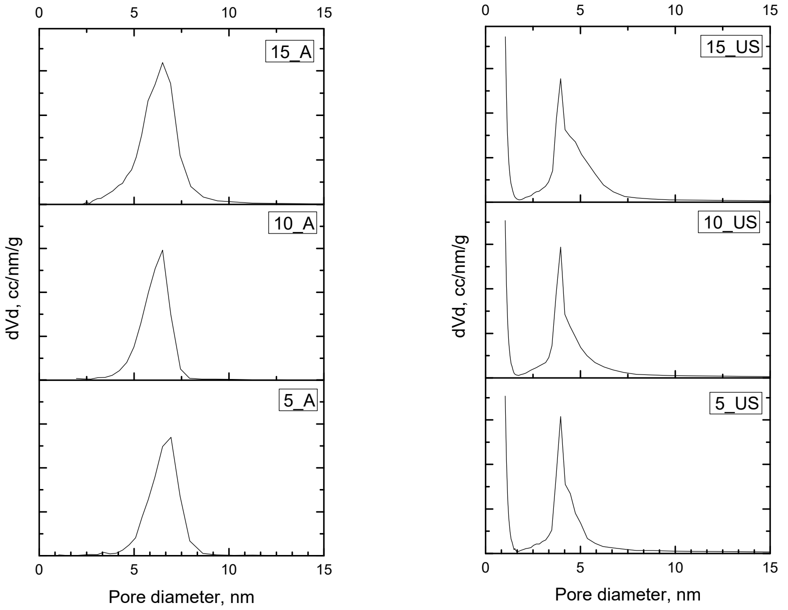

(**a**) (**b**)

Figure S2. Corresponding BJH pore size distributions isotherms for titania samples: (**a**) A-series; (**b**) US-series, calculated using desorption branch of isotherm.
